# Low-quality employment trajectories and the risk of common mental health disorders among individuals with Swedish and foreign background – a register-based cohort study

**DOI:** 10.5271/sjweh.4029

**Published:** 2022-06-30

**Authors:** Roxana Pollack, Bertina Kreshpaj, Johanna Jonsson, Theo Bodin, Virginia Gunn, Cecilia Orellana, Per-Olof Östergren, Carles Muntaner, Nuria Matilla-Santander

**Affiliations:** 1 MRC/CSO Social and Public Health Sciences Unit, Institute of Health & Wellbeing, University of Glasgow, Scotland, UK; 2 Unit of Occupational Medicine, The Institute of Environmental Medicine, Karolinska Institutet, Stockholm, Sweden; 3 Centre for Occupational and Environmental Medicine, Stockholm Region, Stockholm, Sweden; 4 MAP Centre for Urban Health Solutions, Li Ka Shing Knowledge Institute, Unity Health Toronto, Ontario, Canada; 5 Lawrence S. Bloomberg Faculty of Nursing, University of Toronto, Toronto, Ontario, Canada; 6 Social Medicine and Global Health, Department of Clinical Sciences Malmö, Lund University, Sweden; 7 Lawrence S. Bloomberg Faculty of Nursing and Division of Social and Behavioural Sciences, Dalla Lana School of Public Health, University of Toronto, Toronto, ON, Canada

**Keywords:** anxiety, depression, employment trajectory, first generation migrant, migrant mental health, precarious employment, second generation migrant, stress disorder, Sweden

## Abstract

**Objective:**

This study aimed to examine the effects of low-quality employment trajectories on severe common mental disorders (CMD) according to Swedish and foreign background.

**Methods:**

In this longitudinal study based on Swedish population registries (N=2 703 687), low- and high-quality employment trajectories were the main exposures observed across five years (2005–2009), with severe CMD as outcome variable (2010–2017). Adjusted hazard ratios (HR) were calculated by means of Cox regression models and stratified according to Swedish and foreign background [first-generation (i) EU migrants, (ii) non-EU migrants, (iii) second-generation migrants, (iv) Swedish-born of Swedish background] and sex. The reference group was Swedish-born of Swedish background in a constant high-quality employment trajectory.

**Results:**

Second-generation migrants had an increased risk of CMD compared to Swedish-born of Swedish background when following low-quality employment trajectories [eg, male in constant low-quality HR 1.54, 95% confidence interval (CI) 1.41–1.68]. Female migrant workers, especially first-generation from non-EU countries in low-quality employment trajectories (eg, constant low-quality HR 1.66, 95% CI 1.46–1.88), had a higher risk of CMD compared to female Swedish-born of Swedish background. The risk for CMD according to employment trajectories showed little differences between first- and second-generation migrants.

**Conclusion:**

Low-quality employment trajectories appear to be determinants of risk for CMD in association with Swedish or foreign background of origin and sex. Our study shows a higher risk for severe CMD in second-generation and non-EU migrant compared to Swedish-born of Swedish background in constant low-quality employment. Further qualitative research is recommended to understand the mechanism behind the differential mental health impact of low-quality employment trajectories according to foreign background.

Precarious employment (PE) is a social determinant of health and now a highly debated topic of global public health (1). While there is no universally accepted definition, PE is generally conceptualized as a multi­dimensional accumulation of unfavorable employment conditions (2–6). PE has been associated with a variety of health outcomes and suggested mechanisms through which PE may affect health include psychological effects, working hazards, and material deprivation (7–10). PE is more common across individuals in lower socioeconomic positions, lower educational levels, migrants, youth and females (11, 12). In this study, the focus is therefore on low-quality employment by using a multidimensional operationalization and conceptualization of PE. This multidimensional definition includes employment instability (eg, temporary jobs, de-standardized arrangements), lack of power and rights (eg, asymmetric power relations) and poor economic terms (eg, low salary, lack of or minimum training opportunities) (13). Low-quality employment (including PE) has been consistently associated to an increased risk of poor mental health (14–18).

Research on social determinants of mental health has created a growing body of evidence pointing towards the association of precarious employment (PE) with mental health disorders in disadvantaged and marginalized populations (14–16). In 2020, common mental disorders (CMD) were among the main causes of disability-adjusted life years (DALY) globally and the most common work-related health problem in Sweden (19). Associations between depression or anxiety and job loss, unemployment or temporary employment have been found in numerous studies in the last decade, even in countries with universal health care (20, 21).

While differential mental health effects of low-quality employment according to sex have been described, differential effects according to foreign background are understudied. It is of utmost importance to address this knowledge gap for several reasons. First, migrant health needs, especially mental health, are often less visible or neglected and understudied in many areas (22, 23). Second, previous research has found a higher prevalence of mental disorders in both refugees and mixed migrant groups in Sweden (24). Moreover, migrant workers are more frequently found in low-quality employment, and are more vulnerable to labor market marginalization compared to Swedish workers (25). Further, even in the long-term, first- and second-generation migrants are exposed to a range of health risks through post migration factors and the perpetuation of lower socioeconomic positions (2, 26). However, differences between first- and second-generation migrants have been highlighted previously, since the second generation often experiences greater acculturation difficulties, decreased cohesion and poor labor market assimilation depending on the parent generation (27, 28). These complex difficulties for second generation migrants growing up in the host country have been associated with differences in behavioral conditions and higher incidence rates of mental health conditions such as psychosis (29–31). Therefore, it is crucial to account for the diversity in migrant experiences to portray the complex picture of migrant health.

Yet research in this field of study faces several limitations. Previous research has mainly focused on the relation between specific constant employment conditions (such as unemployment or part-time employment) without observing employment changes and adverse mental health outcomes among migrants compared to domestic workers (32). Previous non-register data-based studies on migrant health do not stratify by differentiated migration backgrounds and therefore, lack comparisons between first- and second-generation migrant health effects. Most of the research in this area has used survey-based data, cross-sectional designs, and unidimensional measurements of PE (14, 33, 34). This leaves room for potential bias, reverse causation, misclassification of individuals in long-term exposure analysis as well as insufficient in-depth exploration of the interactions between various components of PE. Moreover, there is a scarcity of research on CMD utilizing trajectory-based approaches to account for an individuals’ dynamic working life course, involving states with various combinations of employment conditions (characterized by low-high quality) as well as transitions between these.

Within this context, the aim of this study is to examine the effects of low-quality employment trajectories on severe CMD according to Swedish and foreign background.

## Methods

### Study design and data collection

This is a longitudinal study based on the Swedish Work, Illness, and Labor-market Participation (SWIP) cohort. This cohort is based on multiple registers that were linked (using personal identification numbers) and anonymized by Statistics Sweden. The annual Longitudinal Integration Database for Health Insurance and Labour Market studies (LISA) was used to retrieve sociodemographic and employment information for all included individuals for 2003–2009 (35). The National Patient Register (NPR) for 1973–2017 was used to obtain diagnoses (ICD-10 codes) and date of admission from inpatient and outpatient care since 2001. Data from a multi-generational register and the NPR were used to retrieve parental data (biological, adoptive) and parental psychiatric history (inpatient data). Data from population census sources for the years 1960, 1970, 1980 and 1990 was used to obtain socio-economic information. Data from the Swedish Cause of Death Register was used to obtain data on death during the study period (36).

As a result of linking registers, the SWIP cohort includes all registered individuals in Sweden – approximately 5.4 million individuals aged 16–65 years – which was then reduced to a subpopulation of N=2 703 687 individuals. The inclusion criteria were (i) residency in Sweden, (ii) having a personal registration number, (iii) being 18–61 years old in 2005 and (iv) participation in the labor market in 2005–2009. Participation in the labor market was operationalized by having an employer and income (except for being unemployed for >180 days, in which case the only requirement was to have an employer during the year of the unemployment). The exclusion criteria were: (i) having died, emigrated, or immigrated in 2005–2009, (ii) having a yearly employment-based income of <1000 Swedish krona in 2005–2009 (not including individuals in unemployment for >180 days), (iii) having a mental disorder diagnosis or having a suicide attempt two years preceding or during the exposure assessment period (2003–2009) (in order to minimize the potential risk of reverse causation), (iv) having incomplete information for measuring the exposure variable, (v) following employment trajectories without any pattern distinguishable into constant, mobility or fluctuating trajectories, (vi) having missing information on migration background (supplementary material, www.sjweh.fi/article/4029, figure S1). The Regional Ethics Board of Stockholm granted ethical permission for the study (no. 2017/1224-31/2 and 2018/1675-32).

### Study variables

*Exposure variable*. The exposure variable used was previously created and complete details can be found in Jonsson et al (37). Briefly, based on the Swedish Register-based Operationalization of Precarious Employment (SWE-ROPE), six employment typologies of lower and higher employment quality were found for 2005–2009: (i) PE relationship (PER), (ii) solo self-employment (SE), (iii) standard employment relationship (SER), (iv) standard employment relationship with high income (SER-HI), (v) business owners (BO), and (vi) hybrid multiple jobholding (HMJH). Additionally, unemployment (UE) was added separately defined by having information on at least one employer and unemployment for >180 days during the year. Then, these employment typologies were grouped into lower (PER, SE, UE) and higher (SER, SER-HI, BO) quality employment. HMJH considered to be of neither lower nor higher quality and was kept separate.

Finally, a total of 21 employment trajectories were manually combined by grouping the combinations of low- and high-quality employment typologies across the five years. Trajectories could follow three different patterns: constant, fluctuating and characterized by mobility. For the purpose of this study, the original 21 employment trajectories were grouped into six broader employment trajectories consisting of: (i) constant high quality: constantly in BO, SER or SER-HI; (ii) constant low quality: constantly in PER, SE, or UE; (iii) mobility high quality: moving upwards from low- to high-quality employment typologies (eg, moving from PER to SER); (iv) mobility low quality: moving downwards from high- to low-quality employment typologies (eg, moving from BO to SE); (v) fluctuating high quality: fluctuating within high-quality employment typologies (eg, moving in and out of BO and SER, BO and SER/HI or SER and SER/HI); and (vi) *fluctuating low quality*: fluctuating within low-quality employment trajectories (eg, moving in and out of UE and BO, UE and SER, UE).

*Outcome variable: common mental disorders (2010–2017)*. The first incidence of CMD documented in inpatient or specialized outpatient care (2010–2017) constitutes the outcome variable. CMD were considered severe, since diagnoses given in non-specialized care were not included. CMD includes diagnoses (ICD-10 codes in brackets) of depression (F32–F33), anxiety (F41), and stress-related disorders (F43). Using severe CMD as an outcome measure allows greater comparability against other register-based studies in both Sweden and countries with similar registers (37). A cumulative measure of these individual diagnoses was chosen due to their shared characteristics, common overlap in individuals with one of these conditions and similar distribution across the study population.

*Stratification variables: sex and background of origin (2005)*. All analyses were stratified according to background of origin to explore if there are differential mental health effects of low-quality employment trajectories according to this variable. We created the variable background of origin based on: “continent of origin” and “Swedish or foreign background”. These considerations were grouped into four categories, based on previous publications (38–41): (i) Swedish-born of Swedish background (born in Sweden, with two domestic-born parents), (ii) second-generation migrants (born in Sweden to at least one foreign-born parent), (iii) first-generation EU migrants from EU-28 countries and Nordic countries, but also including North America and Oceania (with two foreign parents; born-abroad)and (iv) first-generation non-EU migrants from Asia, Africa, South America and the former Soviet Union (with two foreign parents; born-abroad). For conciseness throughout this paper and avoiding small sample sizes in the statistical analysis, first generation migrants from EU-28 countries, Nordic countries, North America, and Oceania have been categorized as “EU” migrants. First generation migrants from Asia, Africa, South America, and the former Soviet Union have been categorized as “non-EU” migrants. While the regions grouped together in both classifications exhibit differences, in general, they share quite many similar characteristics, such as reason and barrier for migration to Sweden.

Moreover, the analyses were also stratified for sex, as many labor markets are still segregated between men and women, and there is evidence of differential mental health effects of low-quality employment according to sex (15, 18, 33).

### Confounding variables

We obtained the minimal sufficient set of variables for adjustment by drawing the causal assumptions using a directed acyclic graph (DAG) (supplementary figure S2) (42). The DAG suggested a minimal sufficient adjustment for estimating the total effect of employment trajectories (2005–2009) on CMD (2010–2017) by using: (i) duration of time since migration (five categories: 1–5, 6–10, 11–20, and 21–59 years, and false coding or missing), which was included to account for potential changes in health status depending on the time spent in the country (43), (ii) age in 2005 (three categories: 18–34, 35–54 and 55–61 years old), (iii) education level in 2005 (three categories: primary/secondary, higher education <3 years, higher education >3 years), (iv) marital status in 2005 (four categories: married/cohabiting with children, married/cohabiting without children, single with children, single without children), (v) parental history of any mental disorder diagnosis or suicide attempt, using the same ICD-codes as above (more than one parent; no parent), between 1964–2004 (before exposure assessment), (vi) parental socio-economic position during the childhood of individuals (occupation type used as proxy, with three categories: manual, non-manual, farmer/self-employed), (vii) previous individual history of any mental disorder diagnosis (ICD-10: F00-F99; ICD-9: 290-316; ICD-8: 290-309) or suicide attempt (ICD-10: X60-X84, Y10-Y34; ICD-9: E950-E959, E980-E989; ICD-8: E950-E959 (yes; no) registered in inpatient between 1964-2002 (before exposure measurement and exclusion criteria). Economic sector and income were not included in the adjustment since they were accounted for in the creation of the employment trajectories.

### Statistical analysis

The accumulated incidence of CMD cases per 100 persons was estimated for 2010–2017, according to employment trajectories grouped by sex and background of origin. Next, we tested the proportionality of hazards using Schoenfeld residuals. After that, we calculated crude and adjusted hazard ratios (HR) with 95% confidence intervals (CI) of CMD by means of Cox proportional hazards regression models. Swedish-born of Swedish background in constant high-quality employment trajectories were used as the reference group. All analyses were stratified by background of origin and sex to account for potentially differential mental health effects of employment trajectories according to these axes of social inequality. One unadjusted model and one confounder-adjusted model were run for the outcome variable. The adjusted model included individual and parental characteristics. The analysis was conducted for both women and men separately, as well as the total population.

Furthermore, three sensitivity analyses were conducted to test the robustness of the adjusted HR. The first model included the number of years since immigration (time of migration) of first-generation migrants to test the potential influence of the time spent in the host country on the HR. The second model excluded individuals in constant unemployment to test a possible increased risk for individuals in this employment status within the constant low quality employment trajectories (44). The last model excluded individuals from former Soviet Union countries from the category non-EU immigrants, because on average, former Soviet Union individuals had higher levels of education and a higher percentage of females when compared with the other categories of non-EU migrants. Further, we ran two logistic regression models, with the outcome common mental disorders and with and without the interaction terms between background of origin and trajectories of quality of employment. We then compared the models with a likelihood ratio test to further test for interaction effects.

Data management and statistical analyses were conducted using STATA version 16 (STATA Corp, College Station, TX, USA) and the figures were created in RStudio using the package ggplot2 (45).

## Results

### Socio-demographic characteristics of the employment trajectories

The distribution of the study population across different backgrounds of origin was 81.5% Swedish-born of Swedish background, 9.1% second generation migrants, 6.5% first generation EU migrants and 2.9% first generation non-EU migrants. The distribution of individuals across the employment trajectories shows that 69.7% of Swedish-born of Swedish background, 66.2% of second-generation migrants, 69.5% of first-generation EU, and 61.9% of non-EU migrants were in constant high-quality employment trajectories. Constant, mobility, and fluctuating low-quality employment were slightly more common among first-generation non-EU migrants compared to other background of origin groups, as shown in table 1.

**Table 1:** Baseline characteristics of the study population (2009) according to background of origin and employment trajectories (2005– 2009) (N=2 703 687). [CMD=common mental disorder; LQ=low-quality; HQ=high-quality].

Employment trajectories (2005–2009)	All	Male	35–54 years old	Primary/secondary education	Prior CMD

	%	%	%	%	%
Swedish-born with Swedish background (N=2 203 149)					
Overall	81.5	53.6	56.7	62.3	2.7
Constant LQ	7.3	62.0	49.1	75.0	3.0
Constant HQ	69.7	51.3	57.5	59.6	2.6
Fluctuating LQ	8.4	57.1	56.5	69.0	3.0
Fluctuating HQ	3.6	65.6	60.5	59.8	2.4
Mobility LQ	4.8	57.4	57.5	68.2	3.0
Mobility HQ	6.3	55.0	55.0	65.4	2.9
Second-generation migrants (N=246 039)					
Overall	9.1	53.9	65.4	62.3	3.4
Constant LQ	8.2	58.4	55.7	72.2	3.9
Constant HQ	66.2	52.1	67.8	59.7	3.2
Fluctuating LQ	9.8	56.8	61.5	68.4	3.8
Fluctuating HQ	3.5	65.2	68.7	59.1	3.0
Mobility LQ	5.5	56.1	63.0	67.5	3.8
Mobility HQ	6.7	53.4	60.2	64.3	3.8
First-generation EU migrants ^a^ (N=17 444)					
Overall	6.4	49.2	59.9	63.3	2.9
Constant LQ	7.6	61.0	58.7	70.0	3.2
Constant HQ	69.5	46.2	59.1	61.7	2.9
Fluctuating LQ	9.0	54.8	63.2	68.6	2.7
Fluctuating HQ	3.1	62.3	63.1	59.4	2.6
Mobility LQ	5.0	54.9	62.5	67.2	3.1
Mobility HQ	5.8	50.1	62.4	64.0	2.8
First-generation non-EU migrants ^b^ (N=80 059)					
Overall	2.9	53.2	66.8	60.9	2.8
Constant LQ	11.2	66.5	61.3	68.8	3.1
Constant HQ	61.9	49.5	68.9	58.5	2.7
Fluctuating LQ	11.1	57.2	63.7	64.0	3.2
Fluctuating HQ	2.9	63.7	69.7	55.7	2.6
Mobility LQ	6.2	54.4	64.7	66.3	2.7
Mobility HQ	6.6	52.8	62.6	61.1	3.0

a First-generation EU countries consist of EU-28, Nordic countries, North America, Oceania.

b First-generation non-EU countries consists of Asia, Africa, South America, former Soviet Union.

### Cumulative Incidence of CMD 2010–2017

Female workers from all backgrounds had a higher incidence of CMD compared to male workers (4.36 versus 2.86). Moreover, first-generation non-EU migrants had the highest CMD incidence compared to other backgrounds for both women (7.28 versus 5.49 for first-generation EU migrants, 4.36 for Swedish-born of Swedish background and 5.29 for second generation migrants) and men (4.14 versus 3.61 for first-generation EU migrants, 2.86 for Swedish-born of Swedish background and 3.27 for second-generation migrants). Next, the incidence of severe CMD was consistently higher within the low- compared to high-quality employment trajectories among both males and females. Among low-quality employment trajectories, the one with the highest incidence of CMD were constant low-quality employment trajectories, except for first-generation non-EU migrants, for which the highest incidence of CMD was found in the mobility low-quality employment trajectories. The group with the highest incidence were women first-generation non-EU migrants in mobility low-quality employment trajectories (8.90) (table 2).

**Table 2:** Common mental disorders (CMD) cumulative incidences per 100 cases (2010–2017) according to employment trajectories by sex and background of origin (N=2 703 687). [CMD=common mental disorder; LQ=low-quality; HQ=high-quality.

Employment trajectories (2005–2009)	Sex			

	Female		Male	
	
	N	Cumulative incidences per 100 cases (2010–2017)	N	Cumulative incidences per 100 cases (2010–2017)
Overall	55061	4.36	41 304	2.86
Swedish-born with Swedish background				
Overall	41 468	4.06	3211	2.72
Constant LQ	3462	5.69	3292	3.31
Constant HQ	27 829	3.72	19 787	2.51
Fluctuating LQ	3903	4.93	3447	3.27
Fluctuating HQ	1038	3.85	1279	2.48
Mobility LQ	2262	5.04	1986	3.28
Mobility HQ	2974	4.77	2319	3.05
Second-generation migrants				
Overall	6007	5.29	4333	3.27
Constant LQ	636	7.56	531	4.49
Constant HQ	3634	4.66	247	2.91
Fluctuating LQ	697	6.68	553	4.03
Fluctuating HQ	158	5.28	171	3.05
Mobility LQ	408	6.83	292	3.82
Mobility HQ	474	6.14	316	3.57
First-generation EU migrants				
Overall	4857	5.49	3097	3.61
Constant LQ	374	7.27	351	4.36
Constant HQ	3342	5.12	1894	3.38
Fluctuating LQ	482	6.79	352	4.09
Fluctuating HQ	107	5.18	112	3.28
Mobility LQ	261	6.59	180	3.73
Mobility HQ	291	5.81	208	4.13
First-generation non-EU migrants				
Overall	2729	7.28	1764	4.14
Constant LQ	266	8.82	270	4.51
Constant HQ	1701	6.79	941	3.84
Fluctuating LQ	328	8.62	234	4.60
Fluctuating HQ	55	6.51	65	4.39
Mobility LQ	203	8.90	128	4.70
Mobility HQ	176	7.04	126	4.50

^a^ First-generation EU countries consist of EU-28, Nordic countries, North America, Oceania.

^b^ First-generation non-EU countries consists of Asia, Africa, South America, former Soviet Union.

### Risk of CMD according to employment trajectories and Swedish or foreign background

**Figure 1:**
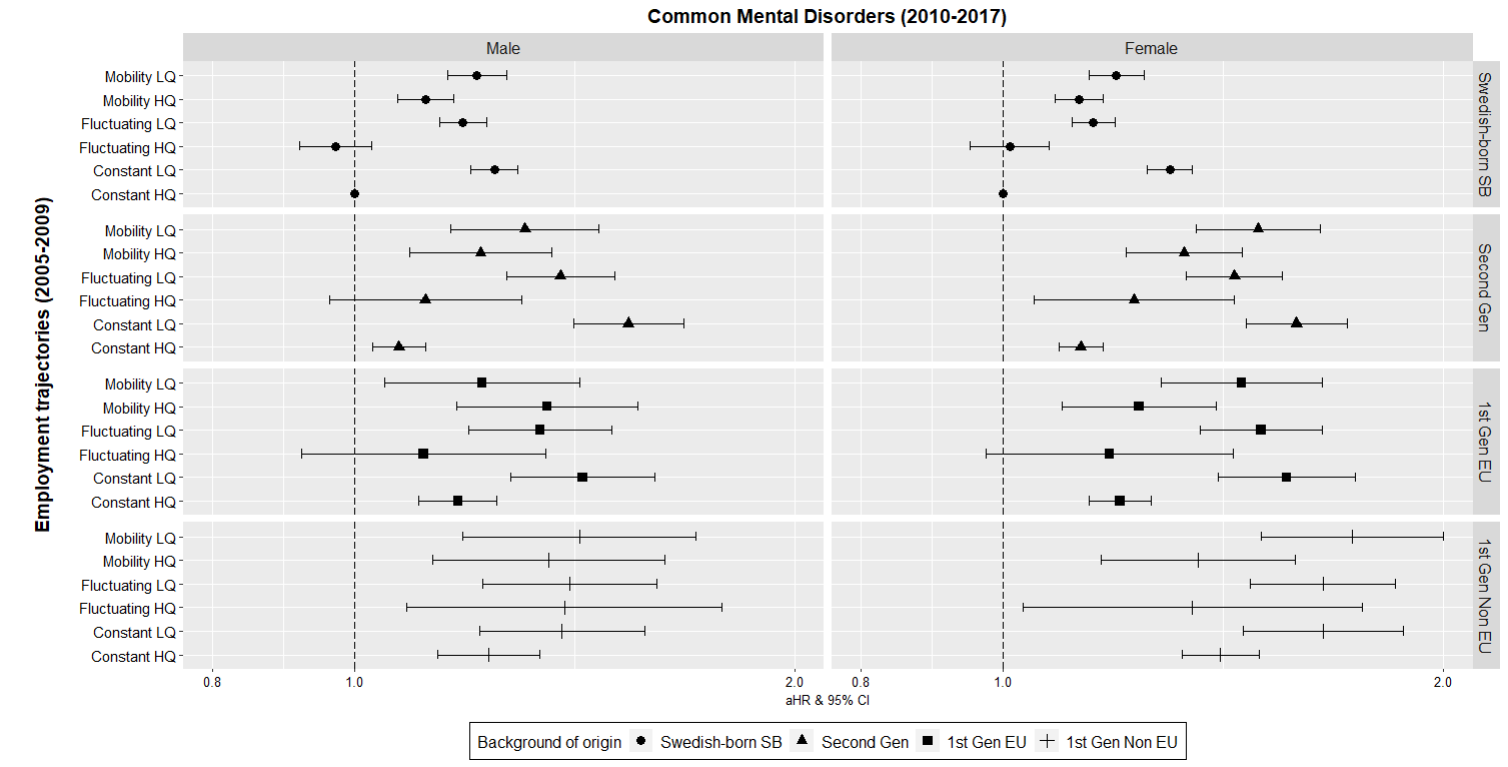
Adjusted hazard ratios (HR) and 95% confidence intervals (CI) for severe common mental disorders (CMD) (2010–2017) according to employment trajectories (2005–2009) and stratified by sex and background of origin (N=2 703 687). The reference group is Swedish born and of Swedish background (Swedish—born SB). The covariates used for adjustment were age, education, marital status, parental mental disorders, parental socioeconomic position, previous mental disorders, duration of time after migration. All covariates have been included in their 2005 version. Second Gen=second generation migrants born in Sweden; 1st Gen EU=first-generation EU migrants (EU-28, Nordic countries and North America, Oceania). 1st Gen Non EU=first generation non-EU migrants (Asia, Africa, South America, former Soviet Union).

While the HR showed clear differences according to background of origin and sex, adjusted estimates showed a higher risk for CMD in all low-quality employment trajectories across all participants with a foreign background and sex (figure 1). Moreover, the magnitude of the HR for all women with a foreign background (first- and second-generation migrants) following low-quality employment trajectories was higher than for female Swedish-born of Swedish background. For example, non-EU female migrants (HR 1.66, 95% CI 1.46–1.88) had about 30% higher risk compared to female Swedish-born of Swedish background (HR 1.30, 95% CI 1.25–1.35) in constant low-quality employment trajectories. Among men, this was also the case but just for second-generation migrants following constant (HR 1.54, 95% CI 1.41–1.68 versus HR 1.25, 95% CI 1.20–1.29) and fluctuating (HR 1.38, 95% CI 1.27–1.51 versus HR 1.19, 95% CI 1.14–1.23) low-quality employment trajectories (supplementary table S2).

After adjusting for all relevant covariates, second-generation male (HR 1.54, 95% CI 1.41– 1.68) and first-generation non-EU female (HR 1.66, 95% CI 1.46–1.88) migrants following constant low-quality employment trajectories had the greatest increased CMD incidence risk estimates when compared to Swedish-born of Swedish background in constant high-quality employment trajectories. The CI of second-generation and first-generation migrants showed a clear differential effect when compared to Swedish-born of Swedish background (male 95% CI 1.20–1.29; female 95% CI 1.25–1.35) in low-quality trajectories.

Within the fluctuating low-quality trajectories, first generation non-EU migrants had the greatest risk of CMD for both sexes (male: HR 1.40, 95% CI 1.22–1.61 and female: HR 1.65, 95% CI 1.48–1.85). The CI of second-generation migrants (95% CI male: 1.27–1.51; female: 1.33–1.55) was significantly different when compared to Swedish-born of Swedish background (95% CI male: 1.14–1.23; female: 1.11–1.19) in this trajectory. Male first-generation migrants showed no distinct differential CI, while EU and non-EU female first-generation migrants also showed differential CMD risks with higher CI than Swedish-born of Swedish background.

In mobility low-quality employment trajectories, first-generation non-EU migrants had the greatest increased risk estimates for both sexes (Male HR 1.42, 95% CI 1.19–1.71; Female HR 1.73, 95% CI 1.50–1.99). Among males, the CI of all background of origin categories overlapped and showed no significant differential effects based on migration background. Among females, second-generation (95% CI 1.35–1.65), first-generation EU (95% CI 1.28–1.65) and non-EU (95% CI 1.50–1.99) migrants overlapped in their CMD effect. However, all first-generation and second-generation migrant female categories had significantly higher risk estimate increases than female Swedish-born of Swedish background in this trajectory (95% CI 1.14–1.23).

The findings are in the line with the tested interaction, which showed interaction effects of background of origin and trajectories of quality of employment on mental health (supplementary table S4). These patterns were similar after excluding former Soviet Union migrants (supplementary table S5) and after adjusting for time since immigration (supplementary table S6). Exclusion of unemployed individuals from constant low-quality trajectories, reduced the effect estimates for female Swedish-born of Swedish background (from HR 1.30 to HR 1.15), but did not change the patterns described (supplementary table S3).

## Discussion

### Main findings

This study has four key findings. Firstly, workers belonging to low-quality employment trajectories showed an increased risk for developing severe CMD in Sweden. Individuals with a migrant background had a greater risk compared to Swedish-born of Swedish background, especially among women. Secondly, male and female second-generation migrants had an increased risk of CMD compared to Swedish-born of Swedish background when following low-quality employment trajectories. Next, female first-generation migrants, especially from non-EU countries in low-quality employment trajectories, had a much higher risk of CMD compared to female Swedish-born of Swedish background. Lastly, the risk for CMD according to employment trajectories showed little differences between first- and second-generation migrants, but both groups exhibited higher risks compared to Swedish-born of Swedish background.

### Interpretation

The findings of this study show differential risk estimates for developing CMD between individuals with and without migrant backgrounds in Sweden belonging to low-quality employment trajectories. This may be explained by several negative migration and protective social factors. For instance, Swedish-born of Swedish background may be benefitting from the absence of negative migration factors (eg, structural and individual level discrimination within socio-economic and political contexts, labor market discrimination, language barriers, lack of social networks) and potential presence of protective societal factors (eg, pre-existing social networks, bonding and bridging social capital, sense of cultural belonging, less stigma attached to mental health) (24, 46).

The results of our study indicate that male and female second-generation migrants had an increased risk of CMD compared to Swedish-born of Swedish background when following low-quality employment trajectories. These results show that despite 74% of second-generation migrants being born in Sweden and with most of them having one Swedish-born parent, second-generation migrants continue to experience negative migration factors (27, 28). Our study findings are supported by previous studies suggesting that second-generation migrant workers are more likely to experience adverse health outcomes compared to non-migrant workers when exposed to poor employment conditions (29, 31). Potential hypothesis to explain these increased mental health effects are exposure to discrimination and racialization in the workplace, followed by continued disadvantages across generations (eg, lack of intergenerational economic mobility and perpetuation of lower socioeconomic positions). In addition, these effects could be exacerbated by divided cultural identities, such as limited sense of belonging or lack of social connection of native born) (27, 28, 47).

Interestingly, we see female first-generation migrant workers, especially from non-EU countries in low-quality employment trajectories, had a much higher risk of CMD compared to female Swedish-born of Swedish background. Previous studies on increased mental disorders risk for migrants in Sweden have suggested that non-EU migrants had an equal or lower risk than Swedish-born of Swedish background due to a potential “healthy migrant effect” for the first 5 years post migration (43). However, our findings support the hypothesis that 10 years post migration these effects most likely faded.

Overall, these findings strengthen the hypothesis that individuals with migrant backgrounds have a higher likelihood of negative mental health effects in adverse employment arrangements when compared to non-migrant workers (43). These results also show greater mental health burden for migrant women. This is in line with previous studies showing that women potentially face greater labor market discrimination in addition to other patriarchal societal and domestic pressures, with a greater negative impact on their mental health (26, 48)

Lastly, the risk for CMD according to employment trajectories showed small differences between first- and second-generation migrants. This seems to suggest a lower impact of low-quality trajectories between different migrant populations themselves. While different acculturation processes and changes in initial health conditions may be responsible for small differences between migrant groups, the risk for developing CMD shows greater similarity within migrant worker groups than between Swedish-born of Swedish background and first- and second-generation migrants.

The findings highlight that, contrary to previous assumptions about advantages enjoyed by EU migrants regarding integration and navigation of healthcare, their background does not seem to benefit their risk for CMD when exposed to long-term PE conditions and acculturation stressors when compared to native-born workers (43, 48).

### Strengths and limitations

The study uses register data, which has high validity and low attrition rates. The large sample size enabled the use of a longitudinal design with an inclusive population (unemployed, self-employed, sex and background of origin-specific analyses). Moreover, the study considered various typologies of employment quality and used a more objective measurement of the outcome and the exposure variable. In this sense, the use of a multidimensional approach better captures the aspects of employment relationships and quality (49). Next, longitudinal trajectories allowed us to capture the dynamic nature of working life. The exclusion of previous mental health disorders before 2003 minimized potential reverse causation.

However, the study is limited by not being able to include refugees or other individuals not registered in Sweden and individuals with mental health issues who have not sought professional care in official institutions or visited primary care. Since we do not have information about primary care visits and only captured the effect of low-quality employment trajectories on severe mental disorders, there also is a possibility for an underestimation of the risk estimates. Next, since some individuals may have changed their low-quality employment status during the follow-up with a potential shift from low quality employment to high quality employment, this could also lead to an underestimation of the effect observed. Also, we were unable to measure all the PE-items initially outlined in Kreshpaj, such as underemployment. Additionally, due to the yearly time-resolution of the data, changes occurring during the year could not be assessed (4, 50).

### Concluding remarks

Low-quality employment trajectories appear to be determinants of poor mental health inequalities in terms of native/non-native background and sex. Second-generation and non-EU migrants following low quality employment trajectories have a higher risk of being diagnosed with CMD compared to Swedish-born of Swedish background following low-quality employment trajectories. Further qualitative research is recommended to better understand the mechanisms behind the differential mental health effects of low-quality employment trajectories in migrants and Swedish-born of Swedish background.

### Ethical statement

The Regional Ethics Board of Stockholm provided ethical permission for this study (no. 2017/1224-31/2 and 2018/1675-32). Funding has been granted through FORTE.

### Funding

The project was funded by FORTE, The Swedish Research Council for Health, Working Life and Welfare, with dnr. 2019-01226.

The authors declare no conflicts of interest or external funding.

## Supplementary material

Supplementary material
