# Supplementary material for: Low-quality employment trajectories and the risk of common mental health disorders among individuals with Swedish and foreign background – a register-based cohort study
Source: Scand J Work Environ Health. 2022 Jun 30;48(5):351–60. doi: 10.5271/sjweh.4029 (PMC9527776; doi:10.5271/sjweh.4029)
Supplement: Supplementary material [file SJWEH-48-351-S001.pdf]

Low-quality employment trajectories and the risk of common mental health disorders among individuals with Swedish and foreign background – a register-based cohort study[1]

1. SUPPLEMENTARY MATERIAL

2. Correspondence to: Roxana Pollack, Unit of Occupational Medicine, The Institute of Environmental Medicine, Karolinska Institutet, Stockholm, Sweden. [E-mail: roxana.pollack@gmail.com]

Table S1. Description of the grouping of trajectories for the exposure variable 'employment trajectories' according to combinations of employment statuses matching different patterns.

Constant trajectories[b]	
Constant low-quality Employment Trajectories	Constant UE [a] (0.75%), Constant SSE (39.84%), Constant PER (51.08%), Constant LQ (8.36%)
Constant high-quality Employment Trajectories	Constant BO (2.44%), Constant HMJH (2.32%), Constant SER (56.16%), Constant SER/HI (31.95%), Constant HQ (7.13%)
Fluctuating trajectories[c]	
Fluctuating low-quality Employment Trajectories	Fluctuating LQ and HM (5.59%), Fluctuating LQ and HQ (92.23%), Fluctuating LQ (0.85%)
Fluctuating high-quality Employment Trajectories	Fluctuating HQ and HM (31.70%), Fluctuating HQ (68.30%)
Trajectories characterized by mobility[d]	
Mobility low-quality Employment Trajectories	Within LQ (0.43%), Between LQ and HMJH (11.78%), Downward HQ to LQ (87.78%)
Mobility high-quality Employment Trajectories	Upward LQ and HQ (74.39%), Within HQ (3.30%), Between HQ and HMJH (22.31%)

a. List of abbreviations: UE=unemployment; PER=precarious employment relationship; SSE=solo self-employment; HMJH=hybrid multiple job holding; BO= business owning; SER= standard employment relationship; SER/HI=standard employment relationship with high income; LQ=low quality; HQ=high quality.
b. Constant trajectories = Spending 4-5 years in a specific employment status, or in combinations of Low Quality or High-Quality employment statuses, independent of order through time.
c. Fluctuating trajectories = Spending a minimum of 2 years in two different employment statuses with fluctuating movement in-and-out of the these. Combinations of LQ- and HQ-employment statuses are also included (if a minimum of 2 years in the LQ- and HQ-classes, respectively).
d. Trajectories characterized by mobility = Spending a minimum of 1 year in one employment status at the beginning of the trajectory, followed by a minimum of 2 years in another employment status at the end of the trajectory. The last 2 years could also be characterized by combinations of LQ- or HQ-employment statuses. Mobility can be upwards, downwards, within or between.

Table S2. Fully adjusted Cox regression model for CMD according to employment trajectories and stratified by Swedish-born with Swedish background and foreign background and sex (n=2,703,687).

	CMD			
	Male		Female	
	aHR[a]	95% CI[b]	aHR[a]	95% CI[b]
Constant HQ	Reference			
Swedish-born with Swedish background				
Constant LQ	1.25	1.20-1.29	1.30	1.25-1.35
Fluctuating HQ	0.97	0.92-1.03	1.01	0.95-1.08
Fluctuating LQ	1.19	1.14-1.23	1.15	1.11-1.19

Mobility HQ	1.12	1.07-1.17	1.13	1.09-1.17
Mobility LQ	1.21	1.16– 1.27	1.20	1.15-1.25
Second-gen migrants				
Constant HQ	1.07	1.03-1.12	1.13	1.09-1.17
Constant LQ	1.54	1.41-1.68	1.58	1.47-1.72
Fluctuating HQ	1.12	0.96-1.30	1.23	1.05-1.44
Fluctuating LQ	1.38	1.27-1.51	1.44	1.33-1.55
Mobility HQ	1.22	1.09-1.36	1.33	1.21-1.46
Mobility LQ	1.31	1.16-1.47	1.49	1.35-1.65
First-gen EU migrants				
Constant HQ	1.18	1.11-1.25	1.20	1.14- 1.26
Constant LQ	1.43	1.28-1.61	1.56	1.40- 1.74
Fluctuating HQ	1.12	0.92-1.35	1.18	0.97- 1.44
Fluctuating LQ	1.34	1.20-1.50	1.50	1.36- 1.65
Mobility HQ	1.35	1.18 - 1.56	1.24	1.10- 1.40
Mobility LQ	1.22	1.05 - 1.42	1.46	1.28-1.65
First-gen non-EU migrants				
Constant HQ	1.24	1.14- 1.34	1.41	1.33- 1.50
Constant LQ	1.39	1.21 - 1.58	1.66	1.46-1.88
Fluctuating HQ	1.39	1.09 - 1.78	1.35	1.03-1.76
Fluctuating LQ	1.40	1.22 - 1.61	1.65	1.48- 1.85
Mobility HQ	1.36	1.13 - 1.63	1.36	1.17-1.58
Mobility LQ	1.42	1.19 - 1.71	1.73	1.50-1.99

a. HR- adjusted Hazard Ratios

b. CI – Confidence Interval

c. Reference group: Swedish born with Swedish background in *Constant high-quality* employment trajectories (n=2,703,687).

Table S3. Adjusted Cox regression model for CMD according to employment trajectories and stratified by background and sex, excluding unemployed individuals in *Constant low-quality* employment trajectories (constant unemployment).

	CMD			
	Male		Female	
	aHR[a]	95% CI[b]	aHR[a]	95% CI[b]
Constant HQ	Reference			
Swedish-born with Swedish background				
Constant LQ	1.24	1.19-1.28	1.15	1.00-1.32

Second-gen migrants				
Constant LQ	1.41	1.28-1.55	1.37	1.26-1.50
First-gen EU migrants				
Constant LQ	1.21	1.07-1.36	1.30	1.17-1.46
First-gen non-EU migrants				
Constant LQ	1.15	1.00-1.32	1.18	1.03-1.35

a. HR- adjusted Hazard Ratios

b. CI – Confidence Interval

c. Reference group: *Constant high-quality* employment trajectories in each stratification group (n=2,703,687)

Table S4. Estimates for risk of CMD according to employment trajectories and background of origin, including an interaction term between employment trajectories and background of origin.

	CMD	
	aOR[a]	95% CI[b]
Employment Trajectories		
Trajectories in Constant high-quality	Reference	
Constant LQ	1.37	1.34-1.41
Fluctuating HQ	0.95	0.91-0.99
Fluctuating LQ	1.30	1.26-1.33
Mobility HQ	1.24	1.21-1.28
Mobility LQ	1.31	1.27-1.36
Background of origin		
**Swedish-born with Swedish background**	Reference	
Second gen	1.22	1.18-1.25
First-gen EU migrant	1.41	1.37-1.45
First-gen non-EU migrant	1.76	1.69-1.83
Background of origin & Employment trajectories		
Swedish-born with Swedish background in Constant HQ	Reference	
Second-gen migrants		
Constant LQ	1.14	1.07-1.22
Fluctuating HQ	1.07	0.95-1.21
Fluctuating LQ	1.08	1.01-1.16
Mobility HQ	1.03	0.95-1.12
Mobility LQ	1.06	0.97 – 1.15

First-gen EU migrants		
Constant LQ	0.94	0.86-1.02
Fluctuating HQ	0.97	0.84-1.21
Fluctuating LQ	0.96	0.89-1.04
Mobility HQ	0.93	0.84-1.03
Mobility LQ	0.89	0.80-1.99
First-gen non-EU migrants		
Constant LQ	0.82	0.74-0.90
Fluctuating HQ	1.02	0.84-1.23
Fluctuating LQ	0.93	0.84-1.02
Mobility HQ	0.86	0.76-0.98
Mobility LQ	0.96	0.85-1.08

a. OR- unadjusted Odds Ratios

b. CI – Confidence Interval

Table S5. Adjusted Cox regression model for CMD according to employment trajectories and stratified by sex among First generation non-EU migrants excluding individuals from former Soviet Union countries. Reference group: *Constant high-quality* employment trajectories (n=2,703,687).

	CMD			
	Male		Female	
	aHR[a]	95% CI[b]	aHR[a]	95% CI[b]
Constant HQ	Reference			
First-gen EU migrants				
Constant LQ	1.15	01.00-1.32	1.20	1.05-1.37
Fluctuating HQ	1.10	0.87-1.43	0.96	0.73-1.26
Fluctuating LQ	1.14	0.98-1.32	1.21	1.07-1.37
Mobility HQ	1.11	0.92-1.34	0.98	0.83-1.14
Mobility LQ	1.15	0.95-1.40	0.98	1.08-1.45

a. HR- adjusted Hazard Ratios

b. CI – Confidence Interval

c. Reference group: *Constant high-quality* employment trajectories in each stratification group (n=2,703,687)

Table S6. Adjusted Cox regression model for CMD according to employment trajectories and stratified by background and sex, including time since migration.

	CMD			
	Male		Female	
	aHR[a]	95% CI[b]	aHR[a]	95% CI[b]
Constant HQ	Reference			
First-gen EU migrants				
Constant LQ	1.21	1.08-1.36	1.30	1.17-1.45
Fluctuating HQ	0.93	0.99-1.13	1.01	0.83-1.23
Fluctuating LQ	1.11	0.98-1.25	1.23	1.11-1.35
Mobility HQ	1.13	1.98-1.30	1.01	0.90-1.15
Mobility LQ	1.02	0.87 – 1.19	1.20	1.05-1.36
First-gen non-EU migrants				
Constant LQ	1.13	0.98-1.30	1.19	1.04-1.36
Fluctuating HQ	1.12	0.87-1.44	0.95	0.73-1.25
Fluctuating LQ	1.14	0.98-1.32	1.19	1.06-1.34
Mobility HQ	1.09	0.90-1.32	0.97	1.83-1.13
Mobility LQ	1.15	0.95-1.39	1.24	1.07-1.44

a. HR- adjusted Hazard Ratios

b. CI – Confidence Interval

c. Reference group: *Constant high-quality* employment trajectories in each stratification group (n=2,703,687).

Figure S1. Flow chart illustrating the study population (2,703,687) after the application of inclusion and exclusion criteria

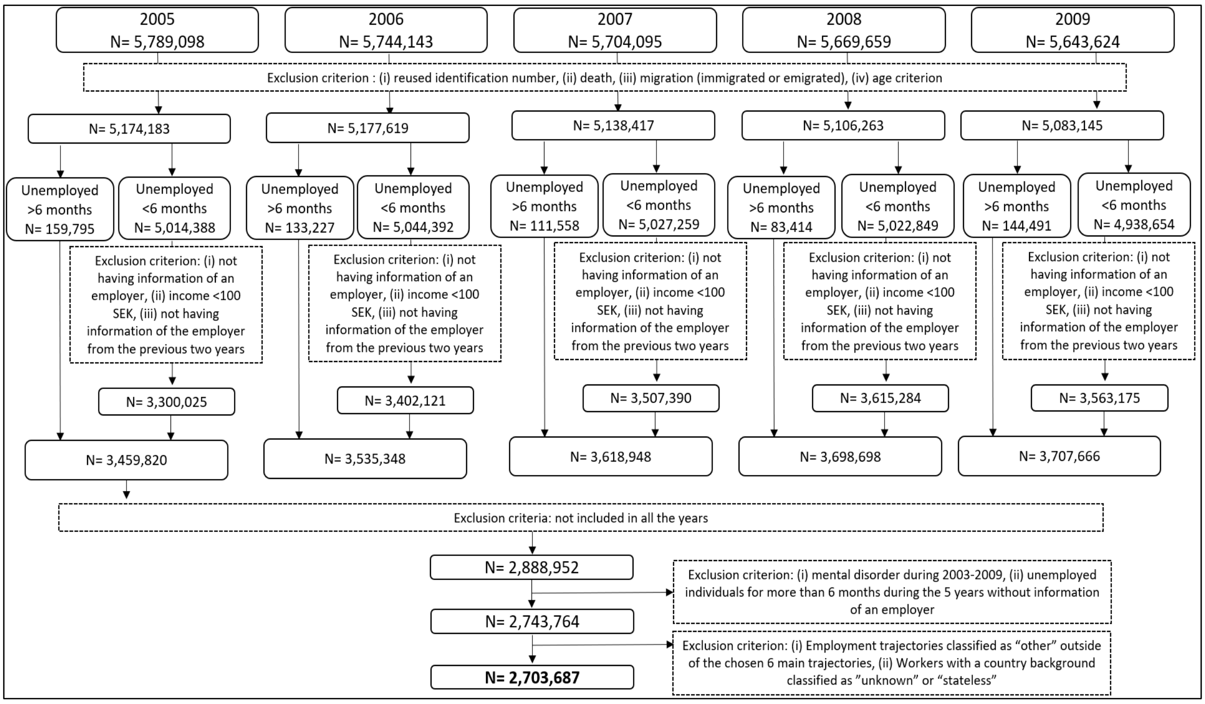

Figure S2. Direct Acyclic Graph (DAG) for the effect of employment trajectories on severe Common mental disorders.

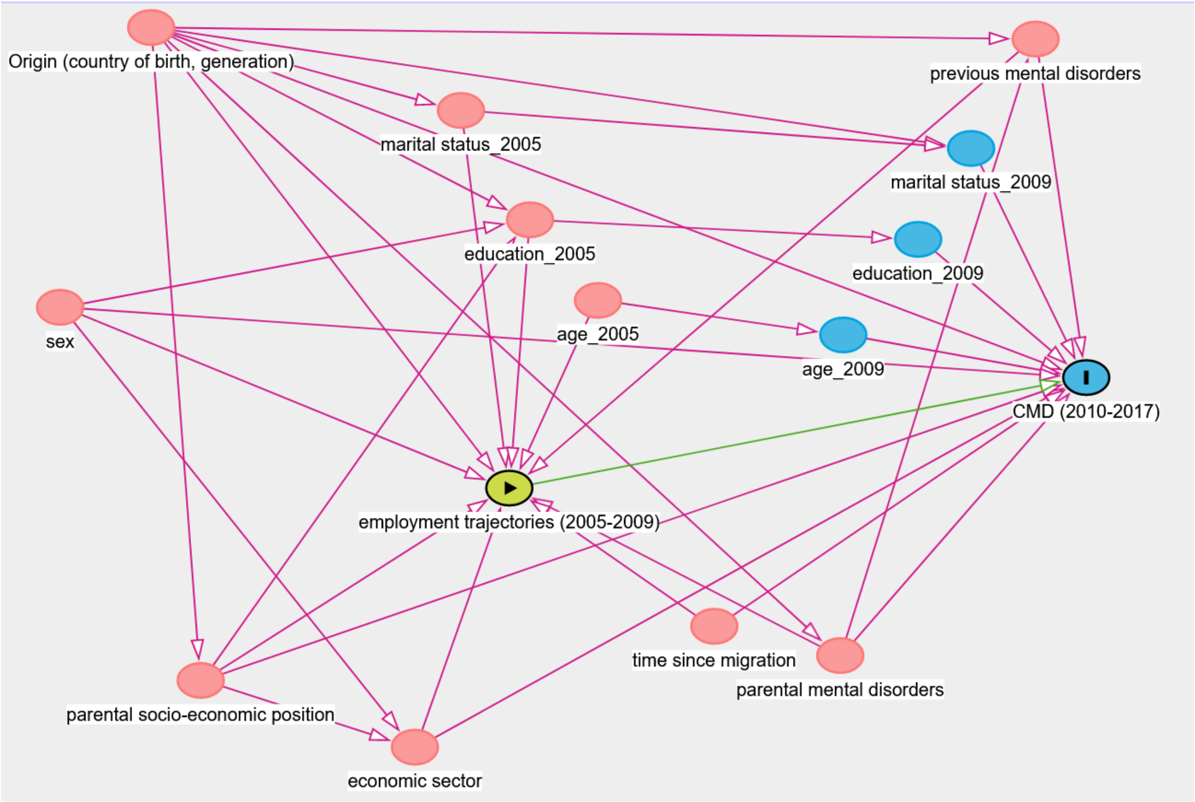

a. Note: The minimal sufficient adjustment sets for estimating the total effect of employment trajectories (2005-2009) on CMD (2010-2017) includes the following variables: Origin (country of birth, generation), age_2005, economic sector, education_2005, marital status_2005, parental mental disorders, parental socio-economic position, previous mental disorders, sex, and time since migration.
